# Ensuring the Correctness and Well Modeling of Intelligent Healthcare Management Systems

**DOI:** 10.1007/978-3-030-51517-1_33

**Published:** 2020-05-31

**Authors:** Samir Ouchani, Moez Krichen

**Affiliations:** 8 Digital Research Centre of Sfax, Sfax, Tunisia; 9 Institut Mines-Télécom, CNRS, Paris, France; 10 Université de Sherbrooke, Sherbrooke, QC Canada; 11 Digital Research Centre of Sfax, Sfax, Tunisia; 12 University of Sfax, Sfax, Tunisia; 13 LINEACT, École d’Ingénieur CESI, 13545 Aix-en-Provence, France; 14 Faculty of CSIT, Al-Baha University, Al-Baha, Saudi Arabia; 15 ReDCAD Laboratory, University of Sfax, Sfax, Tunisia

**Keywords:** Healthcare Management Systems, IoT, UML, RBAC, Formal validation, Alloy

## Abstract

Recent research focus more and more on IoT systems and their applications in order to make people life easier and controllable. The main aim is to expand IoT applications and services into various domains while ensuring communication and automated exchange between them. Recent research handles many issues related to IoT especially implementation, modeling, and deployment. However, many challenges need more deep and thorough analysis especially in terms of flexible modeling, extensible implementation, with respect to the privacy issue. This work focuses principally on modeling IoT systems dedicated to smart healthcare case. We attempt to address the emergency service by initiating a modeling mechanism for Healthcare Management System (HMS) by using UML diagrams, and propose an appropriate access control in order to reinforce it. Then, we ensure the correctness of the developed HMS by relying on the verification and validation based on a formal analysis that showed significant results by using Alloy tool.

## Introduction

Nowadays, unfortunately people are busy and neglect their little health issues such as low pulse rate, high blood pressure, etc. [6]. Among the most challenging objectives of our contemporary society is enhancing healthcare. The provision of quality care to patients while minimizing healthcare costs is a primary goal, while traditional patient evaluation, care, management and supervision practices are often performed manually by nurses [1]. Further, the emergency service is very sensitive for what the patient needs to care in real-time during the treatment period depending the case. Therefor, in order to support and improve healthcare processes we need a flexible system that make the health care services more robust [1, 16] by covering more special cases and respecting safety standards and users/patients privacy.

As evidence, one of the most important priorities of our society is to improve the performance of biomedical systems and healthcare infrastructures [1, 11]. From a research perspective, Internet of Things (IoT) [9, 10] innovations seek to build intelligent systems that support and improve healthcare, by using smart sensors, which allow for automatic monitoring, tracking patients and collecting data from various sources in real-time. Nevertheless, IoT plays an important role in supplying patients and clinicians with convenience in the area of healthcare. This consists of a system which communicates via a network that links internal and external facilities, applications and devices that might support patients and doctors monitor, track and archive critical medical data. Some of the products include wearable health bands, smart meters, and smart video cameras, fitness shoes and smart watches. In this same direction, smartphones applications may contribute in sending medical records and in real time alerts to medical and emergency services [6, 15]. Such interconnected systems create a vast amount of data and information that should be managed effectively. That is already a hard and challenging task to achieve.

In the last few years, many substantial changes in the domain of IoT healthcare are happening. The way human beings and other devices connect and communicate is evolving and becoming better day after day. The ever-growing communication and information technologies [6] allow the control of healthcare outcomes and the decrease of healthcare costs. Consumers, patients and health experts need to think about some creative and more efficient approaches to enjoy the benefits of the revolutionizing IoT in healthcare. With the aid of the ability of IoT, they are now able to gather raw real-time data from very high numbers of patients via smart devices linked to an interconnected network during a continuous period of time. It will take a long time to completely realize the capabilities of these modern technologies. People will see medical experts achieving critical tasks in a much more reliable manner. These experts will not only produce reliable results but they will also save a lot of time, which should be of maximum gain. IoT’s capabilities are really infinite and ever-growing [6].

The basic idea of the proposed solution is to provide patients with reliable and efficient health services by creating a networked knowledge infrastructure so that experts and physicians can make use of this data and make decisions quickly and efficiently. The suggested system will be equipped with apps by which a physician may examine patients anytime from anywhere. It will be also possible to work on emergency scenarios and give a warning to the doctor with the current status of the patient, and complete medical records. We model the whole system by relying on the standard modeling language UML including class, sequence, and use cases diagrams. Further to ensure the privacy of users (practitioners and patients) we develop a role based access control (RBAC) for the proposed health system. To fulfill the healthcare policies and the well correctness of the reinforcement as well as the functionality of the system, the proposed approach relies on Alloy analyzer in order to check automatically the requirements. Alloy [8] can express specifications of a system’s structure and behavior as an abstract model to evolve and expand. Alloy language powered by Alloy Analyzer tool can be used to edit, build, and test its specifications. The analyzer simulates the specification and shows the system’s flaws by checking the correctness and find the counter-examples that help to maintain the system. As well, it has the capabilities to present solutions in graphical format with customizing option. Depending on all these benefits, Alloy has been used for the analysis of many systems including security and software architectures. The proposed solution models first the health care system as well as the reinforcement mechanism using UML and express the related requirements in Alloy. It takes as input the system requirements as assertions and the reinforced model including UML diagrams and RBAC model. The results showed the effective correctness of the modeled system and its related reinforcement mechanism.

The next Sect. 2 describes the related work. Then, we present the modeling and the validation of the system. Finally, Sect. 5 concludes the paper and suggests the possible future research.

## Related Work

In this section, we study the latest IoT contributions, its application in information services, and especially those targeting health care systems. We survey also the contributions dealing with IoT systems modeling, verification and validation.

- Ouchani [14] develops a formal framework to analyze the functional correctness of IoT systems. The proposed framework covers the main components of IoT systems and the approach is automatic. However, the framework suffers from the limitations inherited from PRISM and the security properties are not specified.
- Drira [2] discusses the challenges of modeling IoT in large scale systems called system-of-systems by focusing on interactions and real-time reconfigurations. This contribution presents an overview of architectural concepts without dealing with practical experiments.
- Rahman *et al. *[18] discuss the complexity of distributing services in many IoT devices as well as the challenges facing the system requirements for each level: devices, services, and applications. The proposed model assures a quality attributes such as update, interoperability, security, functional appropriateness, availability and adaptability. However, the verification process suffers from many limitations at the evaluation step.
- Gupta *et al. *[6] design an IoT-based health monitoring framework to reduce the neglect of healthcare. Their solution uses the second generation Intel Galileo board, an Arduino IDE that is explored to program the brain, and Xampp database. This solution provides support emergency medical services by collecting, integrating, and inter-operating of IoT data flexibly. However, they are limited in controlling the access and ensuring privacy.
- Madakam *et al. *[12] review IoT concepts that aim to unify everything into a common infrastructure using smart sensors. First, they focus on basic requirements, geneses, definitions, aliases and characteristics of IoT. Further, they present a various technologies and their usages in innumerable IoT applications into all the domains including medical, manufacturing, transportation, etc. Finally, they acknowledge the need to a standard definition and protocols as well as a universal architecture of IoT that allows for various technologies the ability to inter-operate.
- Gigli *et al. *[5] attempt services categorization provided by IoT systems, which aims to help building a service provider. They consider ubiquitous services which are collaborative aware. However, they require to overcome protocol distinctions among technologies and unify every aspect of the network.
- Mainak *et al. *[13] designs an agent based model which predict the response time of emergency service by taking into consideration the characteristics of road segments and driving behaviour of emergency vehicle drivers. First, they collecting real time driving data by Fire emergency service of Allahabad city using GPS logger HOLUX M1000C. Then, they analyze collected data in GIS along with road network, population density and land use data. Based on the analysis results, they model a fire emergency vehicle (FEV) service. For validation, they compare the theoretical response time with the measured one by simulating scenarios that have 80% matching segments.
- Hussein *et al. *[7] propose a coordination emergency responses framework using agent-based modeling. The main components of this model are Emergency Response Services, Coordination Unit, MCI, Command and Control Center, and Agent Based Simulation. In case of incident, the MCI sends an aid request to the command and control center which transfers all information needed to emergency response services. Next, all resource information are gathered and updated as necessary by this unit. Finally, the resulting plan from the coordination unit will be sent to the agent based simulation, which used to simulate emergency response tasks in real environments, and identify the best coordination mechanism plan to achieve the best response time.
- Catarinucci *et al. *[1] propose a “Smart Hospital System” (SHS) for automatic monitoring and tracking of patients, personnel, and biomedical devices. It composes of a “Hybrid Sensing Network” (HSN) that collects both environmental conditions and patients’ physiological parameters as well as the IoT Smart Gateway that controls the overall SHS behavior. The user interfaces builtin RESTful services which allow user to communicate with the HSN through the 2-way Proxy.
- Molano *et al. *[17] propose an architecture of IoT applied to the industry. First, they present a metamodel that generates industrial cases by extending cyber physical systems to cover covers sensors and actuators to monitor manufacturing. However, safety of data and system accuracy, standardization of technology and interoperability of systems within actual deployments are not considered.
- Cristian *et al. *[4] review the artificial intelligence-IoT fusion with a focus on four important fields. They presents AI basics and the general concepts of computer vision and Fuzzy Logic, and their link with IoT. Besides, they present natural language processing to facilitate the humain-machine interaction.
- Espada [3] proposes a model for constructing and interpreting digital objects in IoT systems which can eliminate the management of pre-configuration and requirements. The model covers the integration and communication of digital objects, applications, devices and users.

Based on the discussed literature, few of them rely on the satisfiability analysis and the stand modeling language to ensure the robustness of the developed solution.

## HMS Modeling

**Fig. 1.**
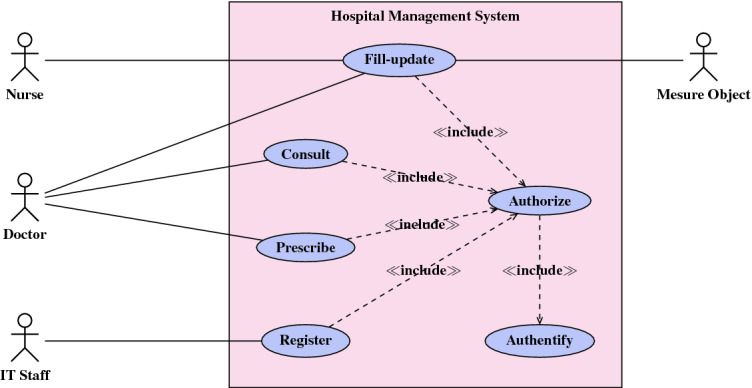
Use case diagram for HMS.

This section describes the structural and behavioral diagrams of **HMS**  proposed system through UML use case, class, and sequence diagrams. Figure 1 shows the main actors and the possible use cases for **HMS**. The doctor can prescribe the status of a patient already registered by the IT Staff, and also can consult his medical reports. Each patient have a medical record, that can be filled or updated by a nurse, a doctor, or a smart object. These cases are only allowed for signed-in and authorized actors. A nurse and smart objects can fill or update the medical record whereas the doctor can consult, fill, update, and prescribe medicines to a patient.

**Fig. 2.**
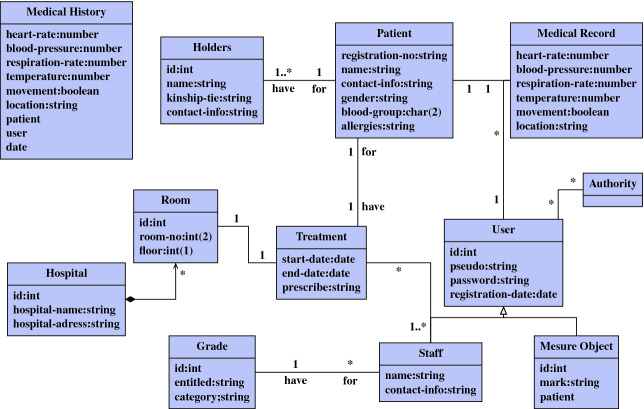
Class diagram for HMS.

Figure 2 shows the main classes that represent the structure of principle users and resources. Only the staff with the IoT can fill or update the patient’s medical record if they have the authority to do that, also each update of the medical record will be saved automatically in the medical history. We consider also to a patient to have a holder regarding his treatments.

## HMS Validation

In terms of system specification, Alloy is a modeling language including a formal syntax and semantics. A specified model in Alloy can be in ASCII format as well with a visual representation. Generally, Alloy targets the formal specification of object oriented data models that can be used generally in data modeling, that also can be displayed graphically. Also for systems analysis, Alloy is a verification tool that automatically analyze the properties (requirements) of alloy models. After checking the properties, Alloy might generate counterexamples in case of the property violation. Alloy consists of predicates, facts, relations and signatures. Signatures represent the different entities of the system. Relations specify the relations between them. Predicates and facts define constraints, which apply on relations and signatures.

Each Alloy model begins with the  declaration. The first step is to declare the signatures using the keyword . Then, we define the relations (fields) which associate atoms. To define a subset, we should use the keywords  or , also the multiplicity keywords such as , , , , etc. Facts correspond to the constraints which must always hold. Finally, we define the predicate using the keyword  and run it.

For the validation of **HMS**, we describe its classes and relations in Alloy specification respecting its syntax and logic. Listing 1.1 shows a fragment of the transformation of **HMS**  into the input language of Alloy where sigs refer to classes, extends to associations, and the multiplicity to the class diagram relations.

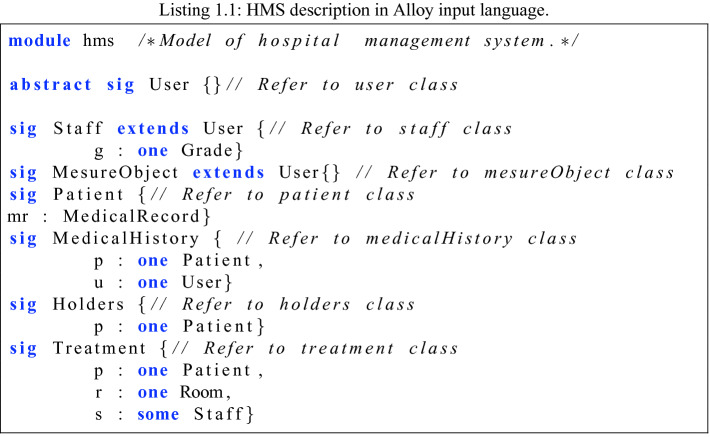



**Fig. 3.**
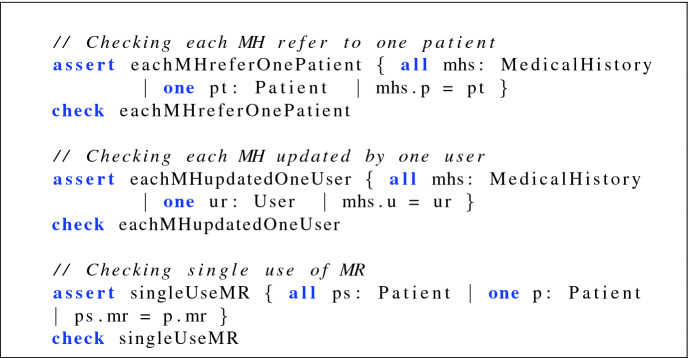
Checking HMS Assertions.

For the effectiveness of the proposed framework, we show the correctness of the proposed model through checking and simulation. We check the assertions related to HMS as described in Fig. 3. The first assertion is to check if only a single user has a medical record. The second looks for the medical history of users whereas the third confirms that the medical data are updated by an authorized user.

## Conclusion

This contribution proposes a concrete model for healthcare system, especially for emergency service in order to facilitate the integration and communication of IoT measures, devices and management systems. First, we proposed UML modeling for the structural and behavioral description of the specific domain of emergency. Further, we proposed Alloy for ensuring the correctness of the model by expressing it safely into its input language. With respect to the obtained results we ensure the correctness of the proposed model. Regarding the obtained results, we target to extend the work in different directions. First, we generalize a meta model from where we can instantiate the model of different services rather than medical and health care. In addition, we have to automate the generation of Alloy code and prove its soundness. Also, we intend to provide, the network architecture including the web services for the different models. Then, we show the impact between them and how we can express and ensure the privacy when deployed. Our final target is to generate a mega system using micro-services architecture, with extensible secure services that can be deployed for different environments.
